# The Indirect Effect of Emotion Regulation on Minority Stress and Problematic Substance Use in Lesbian, Gay, and Bisexual Individuals

**DOI:** 10.3389/fpsyg.2017.01881

**Published:** 2017-10-25

**Authors:** Andrew H. Rogers, Ilana Seager, Nathaniel Haines, Hunter Hahn, Amelia Aldao, Woo-Young Ahn

**Affiliations:** ^1^Department of Psychology, Ohio State University, Columbus, OH, United States; ^2^Department of Psychology, University of Houston, Houston, TX, United States; ^3^Department of Psychology, Seoul National University, Seoul, South Korea

**Keywords:** emotion regulation, lesbian, gay, bisexual, alcohol, substance use, minority stress

## Abstract

Lesbian, gay, and bisexual (LGB) individuals report higher levels of problematic alcohol and substance use than their heterosexual peers. This disparity is linked to the experience of LGB-specific stressors, termed minority stress. Additionally, bisexual individuals show increased rates of psychopathology, including problematic alcohol and substance use, above and beyond lesbian and gay individuals. However, not everyone experiencing minority stress reports increased rates of alcohol and substance misuse. Emotion regulation (ER), which plays a critical role in psychopathology in general, is theorized to modulate the link between minority stress and psychopathology. However, it remains largely unknown whether ER plays a role in linking instances of minority stress with substance and alcohol use outcomes. To address the gap, the current study assessed 305 LGB individuals’ instances of minority stress, ER, and substance and alcohol use outcomes. We assessed the role of ER in problematic alcohol and substance use among LGB individuals using moderated mediation, where sexual minority status was entered as the moderator, and ER difficulties was entered as the mediator. The results indicated significant indirect effects of minority stress, through ER difficulties, on both problematic alcohol and substance use. However, there was no significant interaction with sexual orientation status, suggesting that ER may be important for all LGB individuals in predicting problematic alcohol and substance use. These results highlight the important role that ER plays between instances of minority stress and substance and alcohol use in LGB individuals, suggesting that ER skills may serve as a novel target for intervention.

## Introduction

Lesbian, gay, and bisexual (LGB) individuals experience elevated rates of problematic alcohol and substance use compared to their heterosexual peers (Meyer, 2003; Hatzenbuehler et al., 2008; Bostwick et al., 2010; Coulter et al., 2016; Gillan et al., 2016; Lee et al., 2016; Pakula et al., 2016). Generally, excessive alcohol and substance use can lead to increased incidence of medical problems(Skinner, 1982; Sturm, 2002) and interpersonal problems (Robbins, 1989; Cunradi et al., 2002), as well as high economic cost (Harwood et al., 1998; Sturm, 2002). Researchers have linked the elevated rates of problematic alcohol and substance use observed in LGB populations to sexual minority-specific stressors (e.g., discrimination; Meyer, 2003; Bostwick et al., 2010; Drazdowski et al., 2016). The minority stress theory (Meyer, 2003), a theory central to these health disparities, postulates that LGB individuals experience unique stressors related to their sexual orientation, such as discrimination (e.g., victimization, and family rejection), that increase states of internal distress. LGB individuals then cope with this increase in distress, which is associated with increases in psychopathology, including alcohol and substance use. Importantly, in line with minority stress theory, lower rates of LGB-specific discrimination were associated with decreased rates of problematic alcohol use (Lewis et al., 2016), suggesting that minority stress may be a critical explanatory factor for these elevated rates of problematic alcohol and substance use.

While LGB individuals experience elevated rates of alcohol and substance use compared to heterosexual individuals, differences have been reported within LGB individuals. Bisexual individuals consistently report higher rates of anxiety, depression, and problematic alcohol and substance use compared to their lesbian and gay peers (Koh and Ross, 2006; Bostwick et al., 2010; Pakula et al., 2016). Further, compared to lesbian and gay individuals, bisexual individuals report more current stress, more past adverse events, as well as less support from family or friends (Jorm et al., 2002). These results suggest that bisexual individuals may respond differently to instances of discrimination than to lesbian and gay individuals.

Central to the marginalization of bisexual individuals even within the LGB community may be “bi-phobia.” Indeed, there is research to suggest that both lesbian and gay individuals, as well as heterosexual individuals, may have negative attitudes toward bisexual individuals (Herek, 2002), stemming from both groups challenging the legitimacy of bisexuality as a sexual orientation (Israel and Mohr, 2004). For example, Friedman et al. (2014) suggested that lesbian, gay, and heterosexual individuals characterized bisexual individuals as either confused about their sexual orientation, or lying about it. Bisexual individuals may experience discrimination from both the heterosexual community as well as the lesbian/gay community due to being perceived as both straight and gay (depending on the gender of their partner; Molina et al., 2015). In accordance with the minority stress theory, the additional stigma experienced by the bisexual community acts as a stressor, and coping with these additional stressors likely contributes to the elevated rates of mental health problems (Meyer, 2003; Parnes et al., 2017).

Several studies have examined the effect of discrimination specifically on alcohol and substance use. Researchers have identified LGB-specific family rejection and victimization as specific types of minority stress that are linked to problematic alcohol and substance use (Ryan et al., 2009; Willoughby et al., 2010). Family rejection, defined as a family member’s rejection of an LGB individual’s sexual orientation status (Harter, 1999), may explain why some LGB individuals suffer from elevated rates of psychopathology such as depression and substance use (Ryan et al., 2009; Willoughby et al., 2010; Bregman et al., 2013). Critically, in a study assessing rates of family rejection in LGB adolescents, 36% of respondents indicated experiencing at least one negative reaction from a family member following sexual orientation disclosure (Pilkington and D’Augelli, 1995). Additionally, LGB-specific victimization events, defined as verbal harassment and physical assault due to sexual orientation, affect up to 80% of LGB individuals ages 15–21 (Pilkington and D’Augelli, 1995). This victimization is linked to higher rates of mental health problems in general (e.g., depression, post-traumatic stress disorder; Lee et al., 2016), and problematic alcohol and substance use specifically (Willoughby et al., 2010; Bariola et al., 2016). It is possible that increased negative affect due to elevated rates of discrimination experiences account for the differences in problematic alcohol and substance use between bisexual individuals and lesbian and gay individuals.

Despite evidence linking LGB-specific instances of discrimination with problematic alcohol and substance use, not everyone who experiences discrimination, reports problematic alcohol and substance use. A promising mechanism for this relationship may be regulatory capacity, as a recent study found that LGB individuals who were better able to adaptively cope with LGB-related discrimination showed better overall mental health outcomes (Nadal et al., 2011; Kaysen et al., 2014). In line with this notion, minority stress theory implicates coping mechanisms as a crucial link between experiences of LGB-specific discrimination and negative mental health outcomes (Meyer, 2003). Hatzenbuehler et al. (2009) extended Meyer’s framework by suggesting that coping strategies that regulate affect [emotion regulation (ER)] serve as mediators between experiences of discrimination and psychopathology for LGB individuals (Hatzenbuehler et al., 2009).

Emotion regulation, defined as the process by which individuals alter how they experience and express emotions (Gross, 1998), has been implicated in the onset and maintenance of many psychiatric disorders, including problematic alcohol and substance use (Aldao et al., 2010). Adaptive ER, such as acceptance of emotions, is negatively associated with symptoms of psychopathology (Aldao et al., 2010), whereas maladaptive ER, such as avoidance and rumination, is positively associated with symptoms of anxiety, depression, eating disorders, and alcohol and substance use disorders (Aldao et al., 2010; Shadur and Lejuez, 2015).

A considerable amount of research has linked poor ER to symptoms of psychopathology, particularly problematic alcohol and substance use, yet there is a paucity of work examining these relationships in LGB individuals. Of the available work, ER is related to experiences of minority stress, anxiety, and depression in LGB individuals, with ER difficulties partially explained the relationship between minority status, anxiety, and depression in LGB adolescents (Hatzenbuehler et al., 2008, 2009). Furthermore, previous work has found that LGB individuals often use substances to cope with victimization (Feinstein and Newcomb, 2016). Thus, it is important to further examine ER as a possible explanatory link between experiences of discrimination to problematic alcohol and substance use.

**FIGURE 1:**
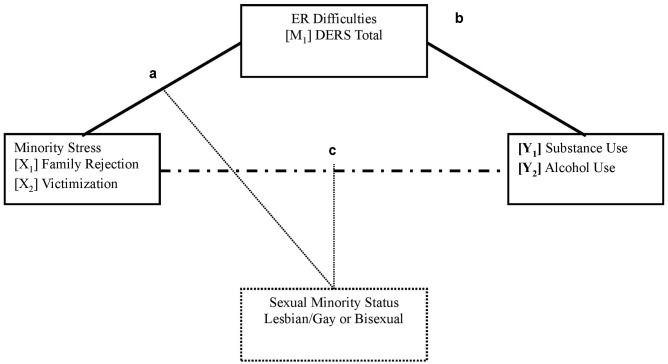
Proposed model. Path “a” describes the effect of minority stress (X) on the mediator (ER difficulties; M). Path “b” describes the effect of ER difficulties (M) on the outcome variables (Problematic alcohol and substance use; Y). Path “c” describes the direct effect of minority stress (X) on problematic alcohol and substance use (Y). The indirect effect of the whole model is the product of path “a” and path “b” (a^∗^b). The moderator (sexual minority status; C), was included as a moderator for the total direct effect, as well as the total indirect effect.

Therefore, to assess the role of ER difficulties on problematic alcohol and substance use among LGB individuals, the current study examined the mediating role of ER difficulties to explain the relationship between family rejection, victimization, and problematic substance and/or alcohol use in LGB individuals. We hypothesized an indirect effect from experiences of discrimination to problematic alcohol and substance use through ER difficulties, where greater levels of minority stress predict greater ER difficulties, which in turn are associated with problematic alcohol and substance use. Additionally, we hypothesize, for ER difficulties total score, the strengths of the associations between the variables will be stronger for bisexual individuals, as more discrimination is likely to lead to increased negative affect, more ER difficulties, and problematic alcohol and substance use. For a visual depiction of the proposed model, see **Figure 1**.

## Materials and Methods

### Participants

Four hundred and ninety two participants were drawn from two online studies (both with identical procedures) examining LGB discrimination and sexual orientation self-disclosure. Recruitment occurred through Facebook advertisements, Craigslist posts, and emails to LGB organizations and listservs recruiting “gay,” “bi,” and “queer” participants for a study on sexual orientation and emotions. Eligible participants needed to: (1) be over the age of 18 years, (2) self-identify as lesbian, gay, or bisexual, and (3) currently reside in the United States. Given the reduced control online data collection affords, we took several measures to ensure maximal data integrity. First, if participants did not meet the three criteria above, the survey automatically closed out preventing the collection of any data. We then kept track of IP addresses in order to ensure that individuals were not answering those questions multiple times until they “met” the inclusion criteria. Second, we excluded participants who provided incomplete surveys (e.g., did not complete any questionnaires), who completed the study multiple times, and who failed more than four “attention questions” designed to catch automated bot programs and disengaged participants (e.g., Oppenheimer et al., 2009; Hauser and Schwarz, 2016). Gender minority (e.g., transgender, gender non-conforming) participants were excluded from the present analyses (*N* = 101) due to sexual orientation-specific stressors likely impacting these participants differently than cisgender participants (Hendricks and Testa, 2012). Additionally, 86 participants were removed due to incomplete survey data, leaving the final study sample with 305 participants (*M_age_* = 28.68, *SD* = 12.08).

### Procedure

After providing informed consent, participants completed a battery of self-report questionnaires. All study participation was completed online via Qualtrics, a web-based survey program that meets high standards for data security^1^. Participation in the entire study took approximately 90 min. Following study completion, participants were debriefed and given a $15 Amazon.com electronic gift card for their participation. This protocol was approved by the Institutional Review Board at the institution where the research was conducted.

### Self-report Measures

#### Alcohol and Substance Use

The *Short Michigan Alcohol Screening Test* (SMAST; Selzer et al., 1975), is a 13-item self-report measure that assesses symptoms [yes (1)/no (0)] of alcohol abuse. Participants are asked if they have experienced a particular alcohol-associated problem (e.g., “Do you ever feel guilty about your drinking”) in the past 12 months. The SMAST has shown strong internal consistency in the current study (α = 0.86). The SMAST total score was computed by adding the total number of problems reported. The *Drug Abuse Screening Test* (DAST; Skinner, 1982), is a 10-item self-report questionnaire that assesses symptoms of substance abuse (e.g., “Have you used drugs other than those prescribed for medical reasons”), excluding alcohol and tobacco on a yes (1)/no (0) scale. The DAST-10 demonstrated strong internal consistency in the present study (α = 0.84). The DAST total score was computed by adding the total number of problems reported.

#### Minority Stress

The *Daily Heterosexist Experiences Questionnaire* (DHEQ; Balsam et al., 2013), is a self-report 50-item measure that assesses emotional distress in response to heterosexist events experienced by lesbian, gay, bisexual, and transgender individuals on a Likert scale from 0 (*Did not happen/not applicable to me*) to 5 (*It happened, and it bothered me extremely*). The present investigation focused primarily on two of the nine subscales that assess family rejection (Family of Origin; e.g., “Being rejected by your mother for being LGBT”) and victimization (e.g., “Being punched, hit, kicked, or beaten because you are LGBT”). Both DHEQ subscales demonstrated strong internal consistency in the present study [α = 0.79 (Family of Origin), α = 0.83 (Victimization)]. Subscales were computed as means of the items for each factor, where the Family of Origin included six items and the Victimization subscale included five items (Balsam et al., 2013).

#### Emotion Regulation

The *Difficulties with Emotion Regulation Scales* (DERS; Gratz and Roemer, 2004), is a self-report 36-item self-report measure that assesses habitual difficulties regulating emotions in a number of dimensions assessed on a 5-point Likert scale from 1 (*Almost never*) to 5 (*Almost always*). The present investigation focused on the DERS total score, which was entered as the mediator in the statistical model sample. The DERS total score was a sum total of all items, and showed excellent internal consistency (α = 0.96).

### Data Analysis

Data analysis was conducted using SPSS. Differences in demographic variables were assessed. Moderated mediation analyses were conducted using the PROCESS macro to determine the conditional effect of sexual orientation status (lesbian/gay vs. bisexual) on the relationship between discrimination and problematic alcohol and substance use, through ER difficulties (Model 8; Hayes, 2013). Bootstrapping with 10,000 data re-sampling was conducted to detect the indirect effects. This method is a non-parametric method used to best estimate the sampling distribution from which the data was collected, which is based on re-sampling the data with replacement (Hayes and Preacher, 2013). A bootstrapped confidence interval that does not include zero is considered statistically significant (Preacher and Hayes, 2008). All mediation analyses controlled for age and gender. Examining missing values using Little’s Missing Completely at Random test indicated that the data were missing at random (*p* > 0.05), and analyses were conducted using listwise deletion, where only those with non-missing data were included in the analyses.

## Results

### Descriptive Statistics

For full descriptive statistics on the sample, see **Table 1**. Independent samples *t*-tests showed a significant difference of age between lesbian/gay and bisexual individuals, where lesbian/gay individuals were, on average, older, (*p* < 0.001), as well as a significant difference for gender, where bisexual individuals were, on average, more likely to be female. The average problematic alcohol use reported for lesbian/gay individuals was 1.56 (*SD* = 2.56), and the average problematic substance use reported for lesbian/gay individuals was 1.23 (*SD* = 1.96). For bisexual individuals, the average reported alcohol use was 1.84 (*SD* = 2.74), and the average reported substance use was 1.68 (*SD* = 2.41). Additionally, there were significant differences on reported victimization, where lesbian and gay individuals reported more severe victimization than bisexual individuals (*p* < 0.001). There were no significant differences for race, ethnicity, education level, family rejection, alcohol use, or substance use between lesbian/gay and bisexual individuals. Correlations between variables are presented in **Table 2**.

**Table 1:** Demographic information for the sample, divided by sexual orientation status.

	Lesbian/Gay (*N* = 190)		Bisexual (*N* = 115)		
			
	*N*	%	*N*	%	*p-*value
Gender (female)	73	38.4	90	78.3	<0.001
Age (years)	30.63	13.26	25.45	8.98	<0.001
Race					>0.05
American Indian/Alaskan Native	5	2.6	1	0.9	
Asian	11	5.8	4	3.5	
Black/African American	7	3.7	3	2.6	
White/Caucasian	156	82.1	100	87.0	
Native Hawaiian/Pacific Islander	0	0	2	1.7	
Other	5	2.6	5	4.3	
Mixed race	6	3.2	0	0	
Ethnicity (non-hispanic)	176	92.6	100	87.0	>0.05
Education					>0.05
Less than high school	1	0.5	0	0	
Some high school	4	2.1	2	1.7	
High school diploma	24	12.6	14	12.2	
Some college	72	37.9	52	45.2	
2-degree/certificate	13	6.8	5	4.3	
4-year college degree	21	11.1	22	19.1	
Some post-graduate	21	11.1	7	6.1	
Post-graduate/professional	34	17.9	13	11.3	


**Table 2:** Bivariate correlations among variables.

	1	2	3	4	5	6	7	8
(1) DAST total	1							
(2) SMAST total	0.538**	1						
(3) DERS total	0.245**	0.186**	1					
(4) Victimization	0.349**	0.311**	0.279**	1				
(5) Family rejection	0.147*	0.170**	0.118*	0.380**	1			
(6) Gender	-0.080	-0.182**	0.170**	-0.154**	-0.077	1		
(7) Sexual orientation	0.100	0.030	0.038	-0.136*	-0.163**	0.387**	1	
(8) Age	-0.021	0.088	-0.254**	0.022	0.094	-0.325**	-0.208**	1


*^∗^*p* < 0.05, ^∗∗^*p* < 0.01.*

### Mediation Analyses

#### Family Rejection and Problematic Alcohol Use

In predicting SMAST total score from family rejection, there was a significant direct effect for bisexual individuals (*B* = 0.94, *SE* = 0.22, *p* < 0.001), but not for lesbian/gay individuals (*B* = 0.04, *SE* = 0.15, *p* = 0.82). Review of the moderated mediation indicated that there was no significant moderation of sexual orientation on the mediational effects of DERS total score (*Index of Moderated Mediation* = 0.02, *SE [Boot]* = 0.06, *Bootstrapped 95% CI* [-0.09, 0.14]). However, results did indicate that there were significant indirect effects of family rejection on SMAST total score, through DERS total score for both bisexual individuals (*B* = 0.09, Bootstrapped 95% CI [0.01, 0.20]), and lesbian/gay individuals (*B* = 0.06, Bootstrapped 95% CI [0.003, 0.16]).

#### Family Rejection and Problematic Substance Use

In predicting DAST total score, family rejection was a significant direct predictor for bisexual individuals (*B* = 0.64, *SE* = 0.18, *p* = 0.006), but not for lesbian and gay individuals (*B* = 0.07, *SE* = 0.13, *p* = 0.61). Review of the moderated mediation indicated that there was no significant moderation of sexual orientation on the mediational effects of DERS total score (*Index of Moderated Mediation* = 0.03, *SE [Boot]* = 0.05, *Bootstrapped 95% CI* [-0.08, 0.14]). However, results did indicate that there were significant indirect effects of family rejection on DAST total score, through DERS total score for both bisexual individuals (*B* = 0.08, Bootstrapped 95% CI [0.01, 0.19]), and lesbian/gay individuals (*B* = 0.06, Bootstrapped 95% CI [0.01, 0.15]).

#### Victimization and Problematic Alcohol Use

For SMAST total score, victimization was a significant direct predictor for bisexual individuals (*B* = 1.82, *SE* = 0.29, *p* < 0.001), but not for lesbian and gay individuals (*B* = 0.31, *SE* = 0.17, *p* = 0.07). Review of the moderated mediation indicated that there was no significant moderation of sexual orientation on the mediational effects of DERS total score (*Index of Moderated Mediation* = -0.05, *SE [Boot]* = 0.05, *Bootstrapped 95% CI* [-0.17, 0.03]). However, results did indicate that there were significant indirect effects of victimization on SMAST total score, through DERS total score for both bisexual individuals (*B* = 0.11, Bootstrapped 95% CI [0.04, 0.23]), and lesbian/gay individuals (*B* = 0.16, Bootstrapped 95% CI [0.07, 0.28]).

#### Victimization and Problematic Substance Use

For DAST total score, victimization was a significant direct predictor for both bisexual individuals (*B* = 1.53, *SE* = 0.23, *p* < 0.001), and for lesbian and gay individuals (*B* = 0.39, *SE* = 0.14, *p* = 0.005). Review of the moderated mediation indicated that there was no significant moderation of sexual orientation on the mediational effects of DERS total score (*Index of Moderated Mediation* = -0.04, *SE [Boot]* = 0.04, *Bootstrapped 95% CI* [-0.15, 0.03]). However, results did indicate that there were significant indirect effects of victimization on DAST total score, through DERS total score for both bisexual individuals (*B* = 0.10, Bootstrapped 95% CI [0.02, 0.21]), and lesbian/gay individuals (*B* = 0.14, Bootstrapped 95% CI [0.06, 0.25]). See **Table 3** for full results and **Figure 1** for the proposed model.

**Table 3:** Direct and indirect mediation results for alcohol and substance use outcomes for bisexual and lesbian/gay individuals.

		*B*	*SE*	*p*-value	95% LLCI	95% ULCI
**Alcohol use**						
**Family rejection**						
Direct effect	Bisexual	**0.94**	**0.22**	**<0.001**	**0.51**	**1.38**
	Lesbian/Gay	0.04	0.16	0.82	-0.27	0.34
Indirect effect	Bisexual	**0.09**	**0.05**	**–**	**0.01**	**0.20**
	Lesbian/Gay	**0.07**	**0.04**	**–**	**0.003**	**0.16**
**Victimization**						
Direct effect	Bisexual	**1.82**	**0.29**	**<0.001**	**1.25**	**2.38**
	Lesbian/Gay	0.31	0.17	0.07	-0.03	0.64
Indirect effect	Bisexual	**0.11**	**0.05**	**–**	**0.04**	**0.23**
	Lesbian/Gay	**0.16**	**0.05**	**–**	**0.07**	**0.28**
**Substance use**						
**Family rejection**						
Direct effect	Bisexual	**0.64**	**0.18**	**0.006**	**0.27**	**1.00**
	Lesbian/Gay	0.07	0.13	0.61	-0.19	0.32
Indirect effect	Bisexual	**0.08**	**0.05**	**–**	**0.01**	**0.19**
	Lesbian/Gay	**0.06**	**0.03**	**–**	**0.01**	**0.015**
**Victimization**						
Direct effect	Bisexual	**1.53**	**0.23**	**<0.001**	**1.07**	**1.98**
	Lesbian/Gay	**0.39**	**0.14**	**0.005**	**0.12**	**0.66**
Indirect effect	Bisexual	**0.10**	**0.04**	**–**	**0.03**	**0.21**
	Lesbian/Gay	**0.14**	**0.05**	**–**	**0.06**	**0.25**


*Direct effects indicate the effect from the type of discrimination to alcohol and substance use outcomes. The indirect effect indicates discrimination on substance use outcome, through DERS total score. All analyses controlled for gender and age. Bolded values indicate statistically significant findings.*

## Discussion

### The Current Study

The goal of the present study was to test whether ER difficulties significantly mediated the relationship between minority status and problematic alcohol and substance use in LGB individuals, and if these effects differed by sexual orientation (i.e., lesbian/gay vs. bisexual). First, in line with previous research (Ryan et al., 2009; Willoughby et al., 2010; Bregman et al., 2013; Bariola et al., 2016), this investigation also found additional support for this link between LGB-related discrimination, and problematic alcohol and substance use, where both victimization and family rejection were significant direct predictors of problematic alcohol and substance use. However, these results differed by sexual orientation for family rejection predicting problematic alcohol and substance use, and victimization predicting problematic alcohol use, where discrimination was a significant direct predictor for bisexual individuals only. The findings suggest that, in line with previous research suggesting bisexual individuals report worse mental health outcomes than lesbian and gay individuals (Koh and Ross, 2006; Bostwick et al., 2010; Pakula et al., 2016), instances of discrimination may have a greater impact on bisexual individuals.

Increases in negative affect are largely associated with poor ER (Aldao et al., 2010). Instances of discrimination, as sources of increased negative affect, are experienced to a greater degree by bisexual individuals (Pilkington and D’Augelli, 1995; Bostwick et al., 2010). It is therefore possible that individuals may use substances as a coping mechanism to decrease their negative affect (Feinstein and Newcomb, 2016), regardless of their ability to regulate negative emotions. ER deficits have been shown to both increase vulnerability for using substances and alcohol to cope as well as developing a substance and/or alcohol use disorder (Shadur and Lejuez, 2015). More research in this area should elucidate the temporal order of discrimination and ER as it related to sexual orientation, as well as the function of alcohol and substance use in the context of minority stress.

Additionally, the present study provided evidence to support ER as an important mechanism explaining alcohol and substance use in response to instances of discrimination. As hypothesized, in the context of family rejection and victimization, overall ER difficulties served a critical role in the pathway between instance of discrimination and problematic alcohol and substance use for LGB individuals. However, contrary to previous research, as well as hypotheses, (Koh and Ross, 2006; Bostwick et al., 2010; Pakula et al., 2016), there no were differences between LGB individuals on the indirect effect of overall ER difficulties. Since victimization (e.g., physical violence, hate crimes, etc.) and family rejection are associated with increases in negative affect (Hatzenbuehler et al., 2009), it makes sense that these instances of discrimination are associated with greater ER difficulties, which in turn is associated with more severe psychopathology for all LGB individuals in the study. Given the emotional intensity of these situations, as well as the strong impetus to find safety, it is possible that ER strategies serve an equally critical role for all LGB individuals.

The findings from this study provide information for novel therapeutic targets for LGB individuals. In line with the model tested in this study, clinicians should assess ER difficulties in LGB clients as a possible mechanism linking instances of discrimination to problematic alcohol and substance use. While no treatments have been specifically developed to target minority stress, ER, and problematic alcohol and substance use in LGB individuals, current treatments targeting ER and problematic alcohol and substance use may have particular utility. For example, Dialectical Behavioral Therapy (DBT; Linehan, 1993) focuses on improving ER capacity in the context of impulsive behaviors, such as alcohol and substance use, often seen in individuals with borderline personality disorder. Other treatments targeting ER difficulties in self-harm behavior (Gratz, 2007) as well as Generalized Anxiety Disorder (Fresco et al., 2013) have shown efficacy, and may be applicable to a population of sexual minorities. Future work should seek to expand these efficacious treatments to LGB individuals to reduce the rates of problematic alcohol and substance use.

### Strengths, Limitations, and Future Directions

This study benefits from a number of strengths, including the large sample size as well as providing initial evidence for ER as a potential mechanism explaining links between LGB-specific discrimination and problematic alcohol and substance use. However, there are limitations that must be noted. First, the data collected is cross-sectional, prohibiting causal and temporal claims to be made regarding the relationships. Additionally, since the study did not explicitly recruit substance and alcohol users, there was limited variability in the scales assessing alcohol and substance misuse. Further, due to the make-up of the sample (primarily white with experiences in higher education), the generalizability of the findings may be limited. Finally, although numerous steps were conducted to minimize lack of control for an online study, it is still possible that participants answered the questionnaires without spending the time to understand the question asked.

Future studies should seek to further elucidate the role that ER plays to explain why LGB-specific discrimination events lead to elevated rates of alcohol and substance misuse. Both laboratory experimental studies as well as longitudinal studies could be used to establish both temporal order of ER, minority stress, alcohol, and substance use, by experimentally inducing minority stress and assessing craving for alcohol and substances. Future research should also more closely examine specific ER strategies that serve as either risk or protective factors for alcohol and substance misuse. Findings from these studies can improve current psychiatric treatments for alcohol and substance misuse, as well as provide culturally competent guidance for therapists working with LGB clients living with alcohol and substance use disorders.

## Conclusion

To our knowledge, this is the first study to examine ER as a mediator between experiences of minority stress and problematic alcohol and substance use in LGB individuals. Results from the study provide initial evidence to support ER as an important mechanism between minority stress, alcohol, and substance use. Results also suggest that assessing and targeting ER in a clinical context with LGB individuals may be important to reduce rates of problematic alcohol and substance use.

## Ethics Statement

All participants provided informed consent, and this study was carried out in accordance with the guidelines set forth by The Ohio State University Institutional Review Board. All subjects gave written informed consent in accordance with the Declaration of Helsinki.

## Author Contributions

AR conducted the data analysis, literature reviews, and wrote the first draft of the manuscript. IS and AA conceptualized the study and collected the data. HH, NH, and W-YA assisted with data analysis as well as provided crucial feedback and edits on the final version of the manuscript. All authors have approved the final version of the manuscript and have agreed to be accountable for all aspects of the work.

## Conflict of Interest Statement

The authors declare that the research was conducted in the absence of any commercial or financial relationships that could be construed as a potential conflict of interest.

## Footnotes

1 www.qualtrics.com/security-statement/

## References

[B1] Aldao A. Nolen-Hoeksema S. Schweizer S. (2010). Emotion-regulation strategies across psychopathology: a meta-analytic review. *Clin. Psychol. Rev.* 30 217–237. 10.1016/j.cpr.2009.11.004 20015584

[B2] Balsam K. F. Beadnell B. Molina Y. (2013). The daily heterosexist experiences questionnaire: measuring minority stress among Lesbian, Gay, Bisexual, and Transgender Adults. *Meas. Eval. Couns. Dev.* 46 3–25. 10.1177/0748175612449743 24058262 PMC3777637

[B3] Bariola E. Lyons A. Leonard W. (2016). Gender-specific health implications of minority stress among lesbians and gay men. *Aust. N. Z. J. Public Health* 40 506–512. 10.1111/1753-6405.12539 27372452

[B4] Bostwick W. B. Boyd C. J. Hughes T. L. McCabe S. E. (2010). Dimensions of sexual orientation and the prevalence of mood and anxiety disorders in the United States. *Am. J. Public Health* 100 468–475. 10.2105/AJPH.2008.152942 19696380 PMC2820045

[B5] Bregman H. R. Malik N. M. Page M. J. L. Makynen E. Lindahl K. M. (2013). Identity profiles in lesbian, gay, and bisexual youth: the role of family influences. *J. Youth Adolesc.* 42 417–430. 10.1007/s10964-012-9798-z 22847752 PMC3500574

[B6] Coulter R. W. S. Birkett M. Corliss H. L. Hatzenbuehler M. L. Mustanski B. Stall R. D. (2016). Associations between LGBTQ-affirmative school climate and adolescent drinking behaviors. *Drug Alcohol Depend.* 161 340–347. 10.1016/j.drugalcdep.2016.02.022 26946989 PMC4792759

[B7] Cunradi C. B. Caetano R. Schafer J. (2002). Alcohol-related problems, drug use, and male intimate partner violence severity among US couples. *Alcoholism* 26 493–500. 10.1111/j.1530-0277.2002.tb02566.x 11981125

[B8] Drazdowski T. K. Perrin P. B. Trujillo M. Sutter M. Benotsch E. G. Snipes D. J. (2016). Structural equation modeling of the effects of racism, LGBTQ discrimination, and internalized oppression on illicit drug use in LGBTQ people of color. *Drug Alcohol Depend.* 159 255–262. 10.1016/j.drugalcdep.2015.12.029 26775286

[B9] Gratz K. L. Roemer L. (2004). Multidimensional assessment of emotion regulation and dysregulation: development, factor structure, and initial validation of the difficulties in emotion regulation scale. *J. Psychopathol. Behav. Assess.* 26 41–54. 10.1023/B:JOBA.0000007455.08539.94

[B10] Gross J. J. (1998). The emerging field of emotion regulation: an integrative review. *Rev. Gen. Psychol.* 2 271–299. 10.1037/1089-2680.2.3.271

[B11] Feinstein B. A. Newcomb M. E. (2016). The role of substance use motives in the associations between minority stressors and substance use problems among young men who have sex with men. *Psychol. Sex. Orientat. Gend. Divers.* 3 357–366. 10.1037/sgd0000185 27713906 PMC5047387

[B12] Fresco D. M. Mennin D. S. Heimberg R. G. Ritter M. (2013). Emotion regulation therapy for generalized anxiety disorder. *Cogn. Behav. Pract.* 20 282–300. 10.1016/j.cbpra.2013.02.001 27499606 PMC4973631

[B13] Friedman M. R. Dodge B. Schick V. Herbenick D. Hubach R. Bowling J. (2014). From bias to bisexual health disparities: attitudes toward bisexual men and women in the united states. *LGBT Health* 1 309–318. 25568885 10.1089/lgbt.2014.0005 PMC4283842

[B14] Gillan C. M. Kosinski M. Whelan R. Phelps E. A. Daw N. D. (2016). Characterizing a psychiatric symptom dimension related to deficits in goal-directed control. *ELife* 5:e11305. 10.7554/eLife.11305 26928075 PMC4786435

[B15] Gratz K. L. (2007). Targeting emotion dysregulation in the treatment of self-injury. *J. Clin. Psychol.* 63 1091–1103. 10.1002/jclp.20417 17932982

[B16] Harter S. (1999). *The Construction of the Self: A Developmental Perspective.* New York City, NY: Guilford Press.

[B17] Harwood H. Fountain D. Livermore G. (1998). *Economic Costs of Alcohol and Drug Abuse in the United States.* Rockville, MD: U.S. Dept. of Health and Human Services.

[B18] Hauser D. J. Schwarz N. (2016). Attentive Turkers: MTurk participants perform better on online attention checks than do subject pool participants. *Behav. Res. Methods* 48 400–407. 10.3758/s13428-015-0578-z 25761395

[B19] Hatzenbuehler M. L. Corbin W. R. Fromme K. (2008). Trajectories and determinants of alcohol use among LGB young adults and their heterosexual peers: results from a prospective study. *Dev. Psychol.* 44 81–90. 10.1037/0012-1649.44.1.81 18194007 PMC3039602

[B20] Hatzenbuehler M. L. Dovidio J. F. Nolen-Hoeksema S. Phills C. E. (2009). An implicit measure of anti-gay attitudes: prospective associations with emotion regulation strategies and psychological distress. *J. Exp. Soc. Psychol.* 45 1316–1320. 10.1016/j.jesp.2009.08.005 20161465 PMC2791410

[B21] Hayes A. F. (2013). *Introduction to Mediation, Moderation, and Conditional Process Anlaysis: A Regression-Based Approach.* New York, NY: Guilford Press.

[B22] Hayes A. F. Preacher K. J. (2013). “Conditional process modeling using structural equation modeling to examine contingent causal processes,” in *Structural Equation Modeling: A second Course*, 2nd Edn, eds Hancock G. R. Mueller R. O. (Greenwich, CT: Information Age Publishing), 219–266.

[B23] Hendricks M. L. Testa R. J. (2012). A conceptual framework for clinical work with transgender and gender nonconforming clients: an adaptation of the Minority Stress Model. *Prof. Psychol.* 43 460–467. 10.1037/a0029597

[B24] Herek G. M. (2002). Heterosexuals’ attitudes toward bisexual men and women in the United States. *J. Sex Res.* 39 264–274. 10.1080/00224490209552150 12545409

[B25] Israel T. Mohr J. J. (2004). Attitudes toward bisexual women and men. *J. Bisex.* 4 117–134. 10.1300/J159v04n01_09

[B26] Jorm A. F. Korten A. E. Rodgers B. Jacomb P. A. Christensen H. (2002). Sexual orientation and mental health: results from a community survey of young and middle-aged adults. *Br. J. Psychiatry* 180 423–427. 10.1192/bjp.180.5.423 11983639

[B27] Kaysen D. L. Kulesza M. Balsam K. F. Rhew I. C. Blayney J. A. Lehavot K. (2014). Coping as a mediator of internalized homophobia and psychological distress among young adult sexual minority women. *Psychol. Sex. Orientat. Gend. Divers.* 1 225–233. 10.1037/sgd0000045 25530980 PMC4267564

[B28] Koh A. S. Ross L. K. (2006). Mental health issues. *J. Homosex.* 51 33–57. 10.1300/J082v51n01_03 16893825

[B29] Lee J. H. Gamarel K. E. Bryant K. J. Zaller N. D. Operario D. (2016). Discrimination, mental health, and substance use disorders among sexual minority populations. *LGBT Health* 3 258–265. 10.1089/lgbt.2015.0135 27383512 PMC4976222

[B30] Lewis R. J. Mason T. B. Winstead B. A. Gaskins M. Irons L. B. (2016). Pathways to hazardous drinking among racially and socioeconomically diverse lesbian women: sexual minority stress, rumination, social isolation, and drinking to cope. *Psychol. Women Q.* 40 564–581. 10.1177/0361684316662603 28138208 PMC5270712

[B31] Linehan M. (1993). *Cognitive-Behavioral Treatment of Borderline Personality Disorder.* New York City, NY: Guilford Press.

[B32] Meyer I. H. (2003). Prejudice, social stress, and mental health in lesbian, gay, and bisexual populations: conceptual issues and research evidence. *Psychol. Bull.* 129 674–697. 10.1037/0033-2909.129.5.674 12956539 PMC2072932

[B33] Molina Y. Marquez J. H. Logan D. E. Leeson C. J. Balsam K. F. Kaysen D. L. (2015). Current intimate relationship status, depression, and alcohol use among bisexual women: the mediating roles of bisexual-specific minority stressors. *Sex Roles* 73 43–57. 10.1007/s11199-015-0483-z 26456995 PMC4594946

[B34] Nadal K. L. Wong Y. Issa M.-A. Meterko V. Leon J. Wideman M. (2011). Sexual orientation microaggressions: processes and coping mechanisms for lesbian, gay, and bisexual individuals. *J. LGBT Issue. Couns.* 5 21–46. 10.1080/15538605.2011.554606

[B35] Oppenheimer D. M. Meyvis T. Davidenko N. (2009). Instructional manipulation checks: detecting satisficing to increase statistical power. *J. Exp. Soc. Psychol.* 45 867–872. 10.1016/j.jesp.2009.03.009

[B36] Pakula B. Shoveller J. Ratner P. A. Carpiano R. (2016). Prevalence and co-occurrence of heavy drinking and anxiety and mood disorders among gay, lesbian, bisexual, and heterosexual Canadians. *Am. J. Public Health* 106 1042–1048. 10.2105/AJPH.2016.303083 26985615 PMC4880251

[B37] Parnes J. E. Rahm-Knigge R. L. Conner B. T. (2017). The curvilinear effects of sexual orientation on young adult substance use. *Addict. Behav.* 66 108–113. 10.1016/j.addbeh.2016.11.012 27918997

[B38] Pilkington N. W. D’Augelli A. R. (1995). Victimization of lesbian, gay, and bisexual youth in community settings. *J. Community Psychol.* 23 34–56.

[B39] Preacher K. J. Hayes A. F. (2008). Asymptotic and resampling strategies for assessing and comparing indirect effects in multiple mediator models. *Behav. Res. Methods* 40 879–891. 10.3758/BRM.40.3.879 18697684

[B40] Robbins C. (1989). Sex differences in psychosocial consequences of alcohol and drug abuse. *J. Health Soc. Behav.* 30 117–130. 10.2307/2136917 2723378

[B41] Ryan C. Huebner D. Diaz R. M. Sanchez J. (2009). Family rejection as a predictor of negative health outcomes in white and latino lesbian, gay, and bisexual young adults. *Pediatrics* 123 346–352. 10.1542/peds.2007-3524 19117902

[B42] Selzer M. L. Vinokur A. van Rooijen L. (1975). A self-administered Short Michigan Alcoholism Screening Test (SMAST). *J. Stud. Alcohol* 36 117–126. 238068 10.15288/jsa.1975.36.117

[B43] Shadur J. M. Lejuez C. W. (2015). Adolescent substance use and comorbid psychopathology: emotion regulation deficits as a transdiagnostic risk factor. *Curr. Addict. Rep.* 2 354–363. 10.1007/s40429-015-0070-y 26889402 PMC4753079

[B44] Skinner H. A. (1982). The drug abuse screening test. *Addict. Behav.* 7 363–371. 10.1016/0306-4603(82)90005-3 7183189

[B45] Sturm R. (2002). The effects of obesity, smoking, and drinking on medical problems and costs. *Health Aff.* 21 245–253. 10.1377/hlthaff.21.2.245 11900166

[B46] Willoughby B. L. B. Doty N. D. Malik N. M. (2010). Victimization, family rejection, and outcomes of gay, lesbian, and bisexual young people: the role of negative GLB identity. *J. GLBT Fam. Stud.* 6 403–424.

